# Supplementary material for: Gut microbiota composition and tumor immune features in meningioma patients
Source: Microbiol Spectr. 2026 Apr 30;14(6):e02485-25. doi: 10.1128/spectrum.02485-25 (PMC13228000; doi:10.1128/spectrum.02485-25)
Supplement: Supplemental figures — Fig. S1 and S2. [file spectrum.02485-25-s0001.docx]

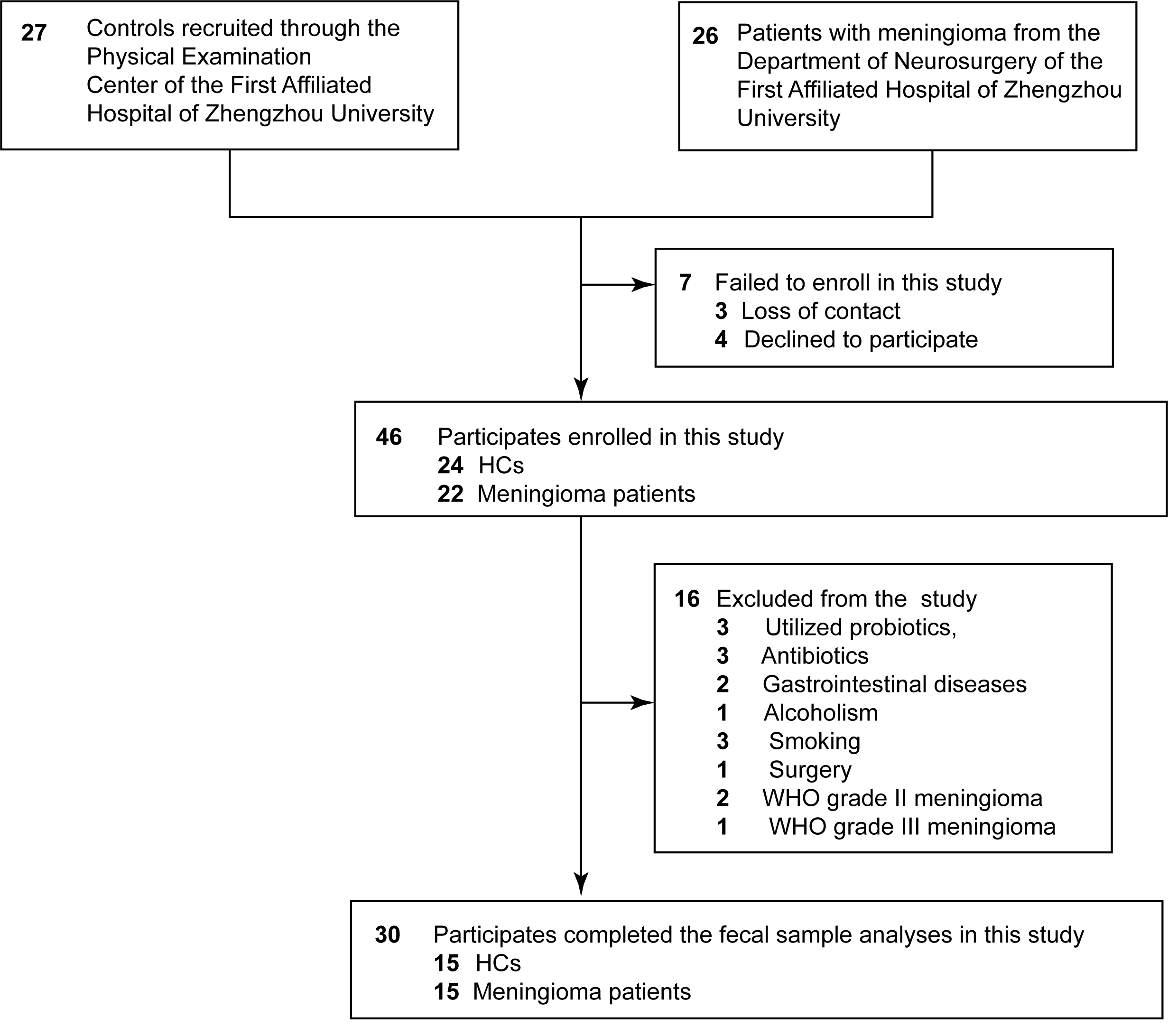


**Supplementary Figure S1. CONSORT diagram.** Consolidated standards of reporting trials flow diagram showing study participants screening, eligibility and inclusion.

***
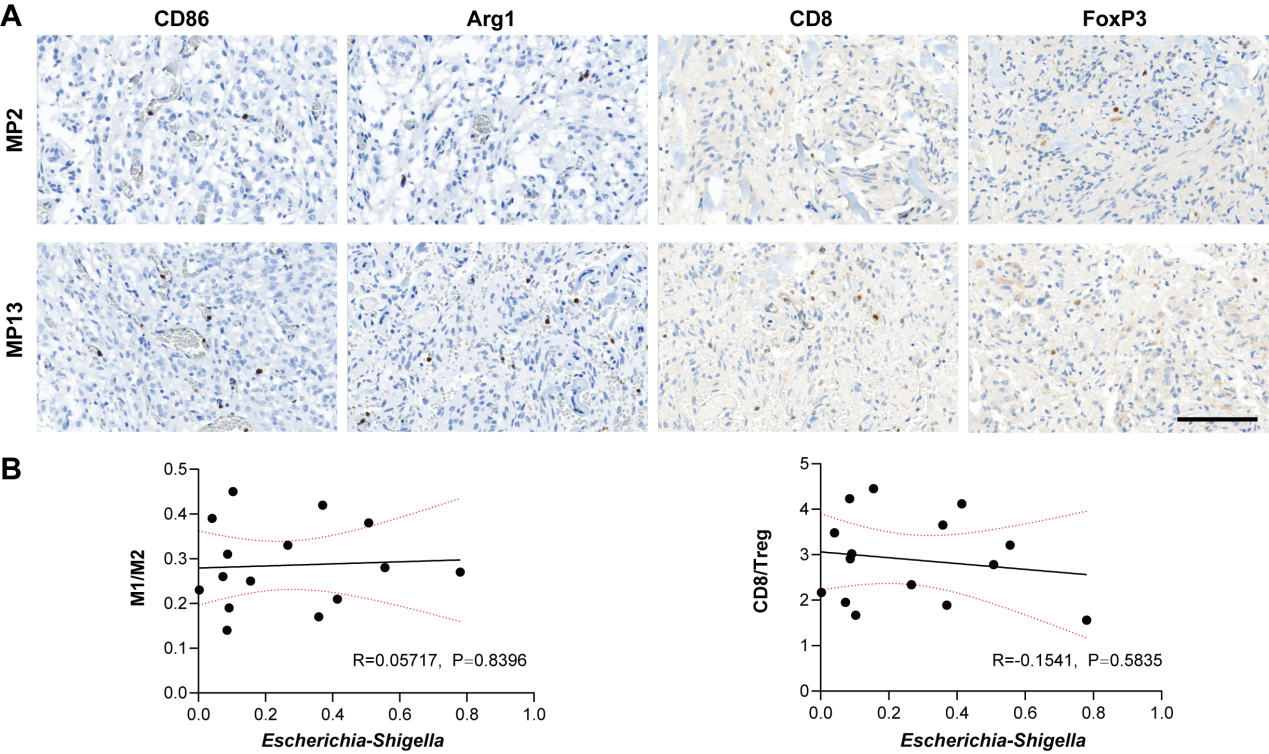
***

**Supplementary Figure S2. Analysis of functional immune cell subsets reveals no polarization associated with Escherichia_Shigella abundance in meningioma.** (**A**) Representative immunohistochemical images of CD86 , Arg1 , CD8 , and FoxP3 in tumor tissues from two meningioma patients with high (MP13) versus low (MP2) fecal Escherichia_Shigella relative abundance. Scale bar = 100 µm. (**B**) Scatter plots showing the correlation between Escherichia_Shigella relative abundance and the ratio of functional immune cell subsets across all patients (n=15).
