# Supplementary material for: Gut microbiota composition and tumor immune features in meningioma patients
Source: Microbiol Spectr. 2026 Apr 30;14(6):e02485-25. doi: 10.1128/spectrum.02485-25 (PMC13228000; doi:10.1128/spectrum.02485-25)
Supplement: Table S2 — Summary of sequencing depth and quality metrics for all samples. [file spectrum.02485-25-s0003.docx]

**Supplementary Table S2.** Summary of sequencing depth and quality metrics for all samples.

| **Sample** | **RawPE** | **Combined** | **Qualified** | **Nochime** | **Base(nt)** | **Avglen(nt)** | **GC** | **Q20** | **Q30** |
| --- | --- | --- | --- | --- | --- | --- | --- | --- | --- |
| HC1 | 127361 | 126264 | 123154 | 114275 | 48101334 | 420.93 | 0.5019 | 0.9829 | 0.9447 |
| HC2 | 104478 | 103928 | 102516 | 96880 | 40712506 | 420.24 | 0.4931 | 0.9871 | 0.9552 |
| HC3 | 95354 | 94931 | 93789 | 90153 | 37727425 | 418.48 | 0.5069 | 0.9894 | 0.9614 |
| HC4 | 154186 | 153387 | 150921 | 149877 | 63020449 | 420.48 | 0.5308 | 0.9879 | 0.9592 |
| HC5 | 104791 | 104334 | 102781 | 96406 | 40143068 | 416.4 | 0.5155 | 0.9886 | 0.9597 |
| HC6 | 105872 | 105450 | 104180 | 63115 | 25868968 | 409.87 | 0.5365 | 0.9896 | 0.9621 |
| HC7 | 153314 | 152502 | 149716 | 145646 | 60651002 | 416.43 | 0.5201 | 0.9862 | 0.9536 |
| HC8 | 98899 | 98516 | 97387 | 72848 | 29843004 | 409.66 | 0.5207 | 0.9903 | 0.9642 |
| HC9 | 78557 | 78271 | 77129 | 71205 | 30183050 | 423.89 | 0.5217 | 0.9878 | 0.9571 |
| HC10 | 103446 | 102907 | 101570 | 94828 | 39671992 | 418.36 | 0.4967 | 0.9888 | 0.9601 |
| HC11 | 79249 | 78887 | 77926 | 72613 | 30399698 | 418.65 | 0.5076 | 0.9889 | 0.9604 |
| HC12 | 88852 | 88109 | 86416 | 86061 | 36328477 | 422.12 | 0.5022 | 0.9869 | 0.955 |
| HC13 | 107939 | 107423 | 105970 | 93016 | 38614321 | 415.14 | 0.516 | 0.9886 | 0.9593 |
| HC14 | 106186 | 105748 | 104336 | 85509 | 35299652 | 412.82 | 0.5272 | 0.989 | 0.9614 |
| HC15 | 135993 | 135076 | 132197 | 115262 | 47964156 | 416.13 | 0.5089 | 0.9841 | 0.9479 |
| MP1 | 145766 | 137199 | 134477 | 121684 | 51456507 | 422.87 | 0.5307 | 0.9827 | 0.9459 |
| MP2 | 104736 | 104285 | 102796 | 94516 | 40018144 | 423.4 | 0.5187 | 0.987 | 0.9552 |
| MP3 | 109034 | 108512 | 107066 | 75546 | 31670894 | 419.23 | 0.5269 | 0.9891 | 0.9604 |
| MP4 | 106088 | 105562 | 104061 | 88727 | 37663077 | 424.48 | 0.5395 | 0.9896 | 0.9619 |
| MP5 | 106006 | 105523 | 103954 | 96538 | 40172469 | 416.13 | 0.5154 | 0.9879 | 0.9568 |
| MP6 | 106542 | 106071 | 104618 | 86188 | 36022273 | 417.95 | 0.5159 | 0.9877 | 0.9565 |
| MP7 | 84886 | 84540 | 83451 | 81165 | 34124549 | 420.43 | 0.5406 | 0.9907 | 0.965 |
| MP8 | 103380 | 102890 | 101591 | 95881 | 40074713 | 417.96 | 0.5256 | 0.9879 | 0.9574 |
| MP9 | 102450 | 101988 | 100786 | 95316 | 39380463 | 413.16 | 0.5159 | 0.9899 | 0.9633 |
| MP10 | 60770 | 60552 | 59833 | 52361 | 21331743 | 407.4 | 0.5337 | 0.9909 | 0.9652 |
| MP11 | 102083 | 101603 | 100090 | 83926 | 34954923 | 416.5 | 0.5163 | 0.9876 | 0.9565 |
| MP12 | 104604 | 104119 | 102671 | 97179 | 40802937 | 419.87 | 0.4957 | 0.9885 | 0.9591 |
| MP13 | 106444 | 105948 | 104484 | 99357 | 42175317 | 424.48 | 0.5217 | 0.9893 | 0.9612 |
| MP14 | 102410 | 101895 | 100569 | 88038 | 37425537 | 425.11 | 0.5104 | 0.9893 | 0.9616 |
| MP15 | 65183 | 64925 | 64110 | 55568 | 23117144 | 416.02 | 0.5334 | 0.989 | 0.9601 |
